# Vacuolar control of stomatal opening revealed by 3D imaging of the guard cells

**DOI:** 10.1038/s41598-023-34273-x

**Published:** 2023-05-11

**Authors:** Filippo Maria Mirasole, Sara Paola Nastasi, Paloma Cubero-Font, Alexis De Angeli

**Affiliations:** 1 IPSiM, CNRS, INRAE, Institut Agro, Université Montpellier, Montpellier, France; 2 Present Address: Department of Plant and Microbial Biology, University of Zurich, Zollikerstrasse 107, CH-8008 Zürich, Switzerland; 3 Present Address: Department of Biosciences, University of Milan, Via G. Celoria 26, 20133 Milano, Italy

**Keywords:** Stomata, Plant transporters

## Abstract

Land plants regulate their photosynthesis and water transpiration by exchanging gases (CO_2_ and H_2_O_vapour_) with the atmosphere. These exchanges take place through microscopic valves, called stomata, on the leaf surface. The opening of the stomata is regulated by two guard cells that actively and reversibly modify their turgor pressure to modulate the opening of the stomatal pores. Stomatal function depends on the regulation of the ion transport capacities of cell membranes as well as on the modification of the subcellular organisation of guard cells. Here we report how the vacuolar and cytosolic compartments of guard cells quantitatively participate in stomatal opening. We used a genetically encoded biosensor to visualise changes in ionic concentration during stomatal opening. The 3D reconstruction of living guard cells shows that the vacuole is the responsible for the change in guard cell volume required for stomatal opening.

## Introduction

Land plants regulate gas exchanges (CO_2_, O_2,_ and H_2_O_v_) through microscopic pores on the leaf surface, the stomata. These pores, delimited by two guard cells, control the diffusion of gases between the leaf and the atmosphere. In all land plants, the two guard cells can actively modify their turgor pressure and shape and thus change the stomatal pore aperture. The guard cell turgor changes depend on the concerted activation of solute (ions and sugar) transporters residing in the plasma membrane, the tonoplast, and other endomembrane systems^1–4^. Several ion transport systems in the plasma membrane (K^+^ and Cl^−^ channels, proton pumps, and sugar transporters) and in the vacuolar membrane (Cl^−^ and malate channels, and H^+^ coupled K^+^, Cl^−^, NO_3_^−^ antiporters) are now identified^5^. These ion transporters and channels are regulated by signaling cascades decoding different stimuli (e.g. light, ABA, CO_2_, humidly, pathogens) and participate in the coordination of the ion transport network of guard cells^2,6^.

Ion transport modifies the osmotic potential of guard cells by inducing massive water fluxes to open and close stomata. The highly efficient intracellular solute transport machinery allows reversible changes of the turgor pressure of the guard cells from 1 to 4.5 MPa^7,8^. Despite these large changes in turgor pressure, the guard cell volume is only modified by 20%^9^ in *Vicia faba*. The combination of turgor pressure and anisotropic properties of the cell wall induces the elastic deformation of the guard cells that regulates the stomatal aperture^10^. Modelling studies have demonstrated the functional links existing between the turgor pressure, the cell wall and the stomatal aperture^10–12^.

During stomatal opening/closure guard cells undergo major and rapid rearrangements of their intracellular organization^13–16^. The cytoskeleton experiences these rearrangements with both the actin and microtubule networks being differently organized in open or closed stomata. Indeed, the cortical microtubules are radially oriented in open stomata but not in closed ones^17,18^. However, in guard cells the more dramatic intracellular modification concerns the vacuole morphology^13,15,16^. Indeed, the vacuole is deeply reorganized, with both its volume and structure being rapidly modified. In closed stomata, the vacuole is fragmented in several vesicles that can be interconnected^13,19^. In contrast, in open stomata the vacuole is a single compartment occupying a large proportion of the whole cell volume^15,16^. The vacuole stores the major part of the solutes accumulated by the guard cells and is therefore central for the control of the turgor pressure. Indeed, the solute concentration in guard cells reaches the molar range, a concentration too high to be tolerated by the cytosol^8,20^. The vacuolar expansion and shrinking relies on its capacity to accumulate ions^15^. In parallel to the vacuole, the nuclear and cytosolic compartments also reorganize within the limited space of the guard cell to accommodate the vacuolar rearrangements during stomatal movements. However, surprisingly the nuclear and cytosol dynamics in the guard cells are almost unknown. Further, in guard cells the functional connection between the subcellular organization and the ion transport machinery of the cellular membranes is still poorly understood.

Here we investigated in *Arabidopsis thaliana* guard cells, from a quantitative point of view, the link between ion transport reactions and the rearrangement of subcellular compartments. We therefore developed a strategy based on a genetically encoded biosensor and 3D imaging to follow, in living guard cells, pH_cyt_ and [Cl^−^]_cyt_ during stomatal opening in parallel with the volumetric changes of the subcellular compartments. We quantified, in individual guard cells, the volume and the morphological modifications of the vacuolar and cytosolic compartments together with the pH_cyt_ and [Cl^−^]_cyt_ dynamics. Surprisingly, our data show that the cytosolic volume is constant and that the vacuole is responsible of the guard cell volume changes. Consequently, our data show that the morphological and volume modifications of the vacuole are the processes driving stomatal opening.

## Results

### Cytosolic pH and [Cl^−^] dynamics in fusicoccin-induced stomatal opening.

**Figure 1:**
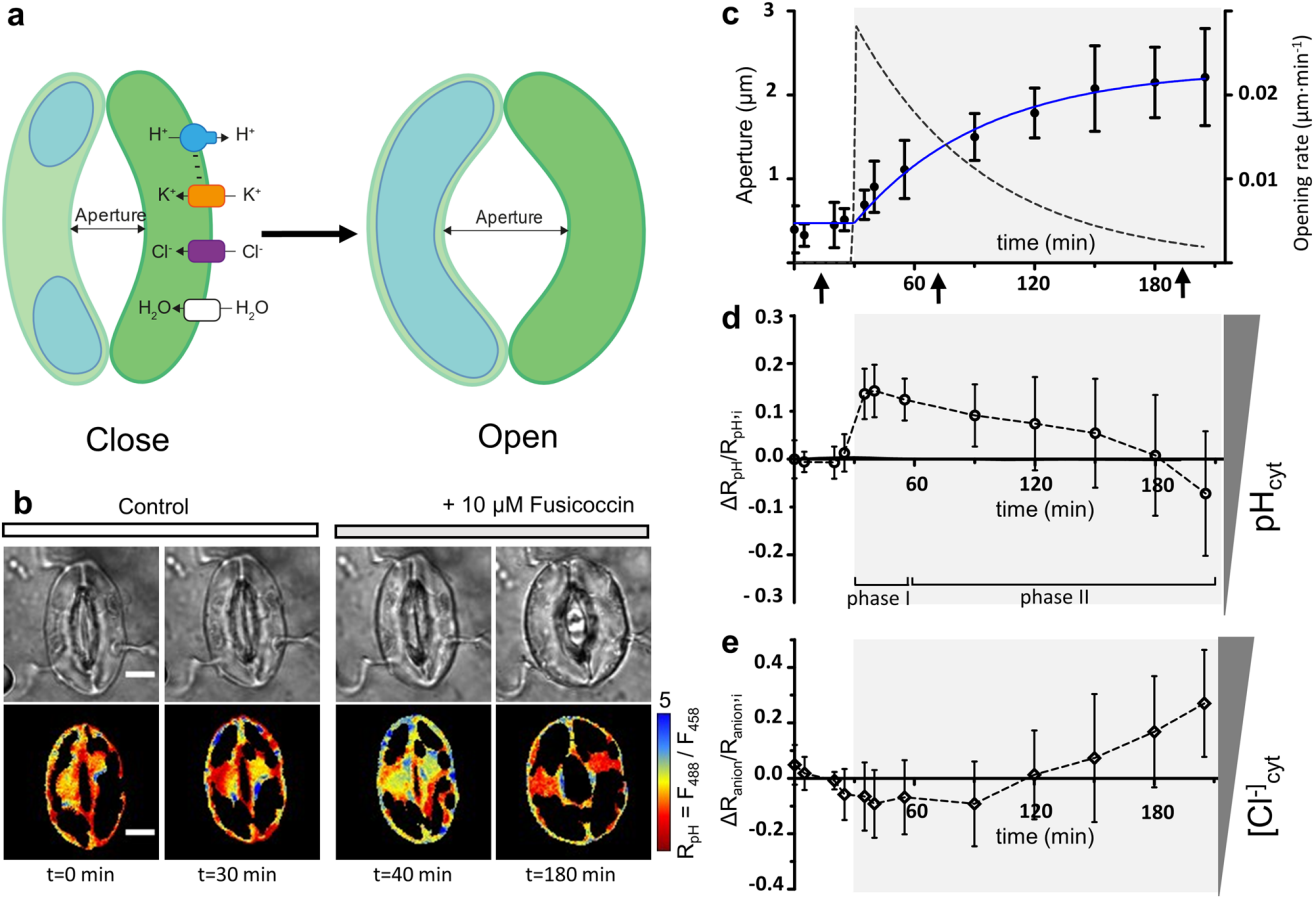
pH_cyt_ and [Cl^−^]_cyt_ dynamics during stomatal opening. **(a)** Schema of fusicoccin-induced stomatal opening. fusicoccin directly activates the plasma membrane H^+^-ATPases inducing extrusion of H^+^ to the apoplast and a hyperpolarization of the plasma membrane. This induces the influx of solutes (i.e. K^+^ and Cl^−^) inside the guard cell, decreasing its water potential and driving the influx of water. (**b**) Representative transmitted light (top) and false color images of R_pH_ (bottom) representing pH_cyt_ in a stomata expressing ClopHensor at different time points before (control) and after fusicoccin-induced opening. The black space within the guard cells in the R_pH_ images represents the vacuole. During the whole experiments stomata were exposed to 0.1% DMSO. Scale bars 5 µm. (**c**) Time resolved stomatal aperture kinetics over 3 h, and after application of fusicoccin (grey area, n = 11 stomata). Data were fitted (solid blue line) with plateau followed by an exponential function. Opening rate was calculated as the first derivative of the exponential fit (black dotted line). Arrows indicate the time points corresponding to the acquisition for 3D reconstruction (see Fig. 3). (**d**) Time resolved pH_cyt_ changes represented as *ΔR*_*pH*_*/*_*RpH,i*,_ (n = 11 s stomata) during stomatal opening (*grey area*). After fusicoccin application pH_cyt_ rapidly increased before decreasing to values close to the initial ones. (**e**) Time-resolved changes of [Cl^−^]_cyt_ represented by *ΔR*_*anion*_*/R*_*anion*_*,*_*I*_ (n = 11 stomata). After fusicoccin (grey area) *ΔR*_*anion*_*/R*_*anion*_*,*_*I*_ was stable for the first 60 min. After it raised, indicating an increase of [Cl^−^]_cyt_. In (**c–e**) data are presented as mean ± S.D.

We induced stomatal opening under a confocal laser scanning microscope with fusicoccin. This fungal toxin activates the plasma membrane proton pumps, stimulating ion fluxes directed into the guard cells^21^ (Fig. 1a). We designed stomatal opening experiments using *Arabidopsis thaliana* plants expressing the biosensor ClopHensor to measure the changes of pH_cyt_ and [Cl^−^]_cyt_^22^ (Fig. 1). ClopHensor allows simultaneous visualization and quantification of pH_cyt_ and [Cl^−^]_cyt_ in living guard cells (Fig. 1). To start the stomatal opening experiments with guard cells in a defined physiological state, Arabidopsis leaf epidermal peels were prepared at the end of the dark period (i.e. 1 h before light onset), when stomata were still closed (Fig. 1b). Firstly, stomata were imaged for 30 min in a control buffer with 30 mM KCl (see “Material and methods”). Then, 10 µM fusicoccin was applied and the stomatal opening process was followed for a total of 195 min (Fig. 1b–e). The stomata opened to a pore width of 2.2 ± 0.6 µm (n = 9 stomata) after 110 min in fusicoccin (Fig. 1c and Fig S1). The stomatal aperture kinetics were fitted with a plateau followed by an exponential function and we estimated an opening half-time of about 43 min that is in the same range of those observed in whole leaves after exposure to light^23^ (Fig. 1c). Notably the stomatal opening rate was maximal just after fusicoccin application and afterwards decreased over time (Fig. 1c).

In the same stomata the fluorescence of ClopHensor was imaged after excitation at 488 nm, 458 nm and 561 nm. These data were used to calculate the two ratios: R_pH_ (F488/F458) and R_anion_ (F458/F561), which report pH_cyt_ and [Cl^−^]_cyt_, respectively^22^ (Fig. 1b). ClopHensor is sensitive to Cl^−^ and NO_3_^−^, with different affinities^22,24^. In the present set of experiments, we used a buffer based on 30 mM KCl and, therefore, R_anion_ variations can be interpreted as [Cl^−^]_cyt_ changes. After application of fusicoccin, pH_cyt_ rapidly increased, reaching a maximum within 5–10 min (Fig. 1d). Faster image acquisition confirmed that pH_cyt_ maximum was within the 5 min after fusicoccin application (Fig. S2). This behaviour of pH_cyt_ was previously observed^22^ and shows that, when the stomatal pore is still closed, fusicoccin rapidly activates the plasma membrane H^+^-ATPases inducing H^+^ extrusion and a rapid pH_cyt_ increase. In a second step, pH_cyt_ decreased and reached its initial values (Fig. 1c, d). This phase coincides with the maximum pore aperture. In contrast, [Cl^−^]_cyt_ was mostly constant in the first 60 min after fusicoccin application (Fig. 1e). Only after this period, a raise of [Cl^−^]_cyt_ was observed (Fig. 1e). Interestingly, the [Cl^−^]_cyt_ increased only when the stomatal pore aperture was above the half of its maximum, i.e. when the turgor pressure of the guard cell was maximal^10^. Anions like Cl^−^ significantly contribute to the osmotic potential of guard cells^8^, explaining why Cl^−^ concentration and pore aperture maximum coincided and, consequently, the turgor pressure too.

### 3D reconstruction of the vacuole with a “negative staining” approach

**Figure 2:**
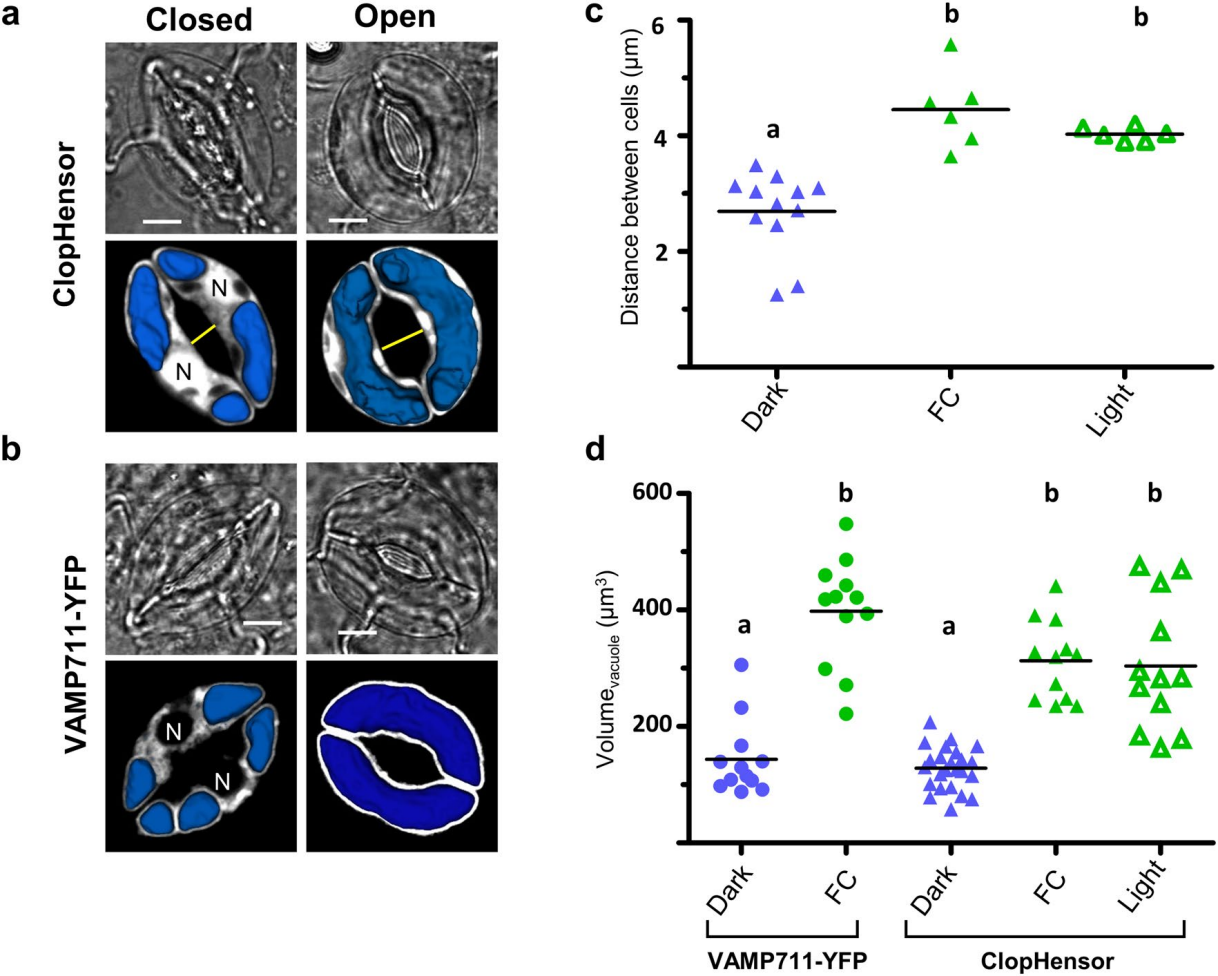
3D reconstruction of vacuole in guard cells.** (a,b)** Representative transmitted light images (top) and confocal images with superimposed reconstructed vacuolar 3D models (bottom) of guard cells. (**a**) Guard cells expressing ClopHensor in the cytosol and the nucleus. (**b**) Guard cells expressing the tonoplast marker VAMP711-YFP. In (**a,b**) images were obtained after incubation in darkness for 180 min in control buffer (left column, closed) and in presence of 10 µM fusicoccin (right column, open). Scale bars 5 µm. (**c**) Distance between guard-cells (yellow line in **a,b**) of each stomata measured from the middle fluorescence *z*-stack image with or without fusicoccin. Samples were left 180 min under white light (n = 6 stomata, open green triangles), fusicoccin (n = 6 stomata, filled green triangles) and in darkness (n = 6 stomata, filled blue triangles). (**b**) Vacuolar volumes calculated from the 3D models of guard-cells expressing VAMP711-YFP (circles) and ClopHensor (triangles). Blue symbols, samples kept in dark for 180 min (n = 12–24 guard cells); green symbols, samples kept in the dark with10 µM fusicoccin for 180 min (n = 12 guard cells); open triangle samples exposed for 180 min to light (n = 12 guard cells). Statistical significance was determined with a one-way ANOVA with non-parametric Dunnett’s post-hoc test, letters indicate significant differences (p < 0.01). (**c,d**) Horizontal line in the dot plots indicates the mean.

The ratiometric images of the stomata obtained at different time points suggest that the relative importance of the nucleus-cytsolic and of the vacuolar compartments varies during stomatal opening (Fig. 1b). In these 2D images the cytosol appears to undergo a size reduction while the vacuole increases. To better study these changes, we quantified the volume of the vacuolar compartment of guard cells during stomatal opening (Fig. 2). Indeed, in guard cells, the vacuole is the subcellular compartment undergoing the major modifications during stomatal opening/closure^13,15,16^. Notably, the morphological modifications of vacuoles have been investigated in many plant cell types^19,25^. However, in guard cells no quantitative volumetric changes of both the vacuole and of the nuclei-cytosolic compartment have been reported so far. To tackle this issue, we used a 3D reconstruction approach mixing “direct and negative staining” of the subcellular compartments by ClopHensor. Indeed, ClopHensor expression in both the cytosol and the nucleus directly labels these compartments, and it additionally provides a “negative staining” of the vacuole (Fig. 2). Thus, the vacuole can be then identified as the intracellular region where no fluorescent signal of the E^2^GFP moiety of ClopHensor is visible, i.e. “negative staining” (Fig. 2). This strategy enables the quantification of the volumes of the two major compartments in a single guard cell during stomatal opening. Thus, to test this possibility we quantified the vacuolar volumes obtained from guard cells expressing ClopHensor and from guard cells expressing the tonoplast marker YFP-VAMP711^25^ (Fig. 2). Epidermal samples were prepared at the end of the dark period, as previously described. The samples were either kept in the dark, or exposed to fusicoccin 10 µM or to light for 3 h (Fig. 2a,b). We acquired z-Stacks of stomata in the different treatments exciting at 488 nm the E^2^GFP moiety of ClopHensor^22,26^, or the YFP in VAMP711-YFP (Fig. S3). The z-Stacks were then manually segmented using 3D Slicer software (https://www.slicer.org)^27^ to reconstruct the vacuole in each guard cell (see “Material and methods”).

In closed stomata the vacuole was fragmented in guard cells expressing both ClopHensor (n = 24 guard cells) and VAMP711-YFP (n = 12 guard cells) (Fig. 2a,b). The vacuolar compartment was divided in multiple vesicles of different sizes (Fig. 2a, b). Interestingly, in all cases we observed the presence of two major vacuolar vesicles systematically localized at the two poles of each guard cell (Fig. 2a,b). The nuclear region was in the central area of the guard cell separating the two polar vacuolar vesicles (Fig. 2a,b). In the guard cells expressing VAMP711-YFP, we observed fluorescence in the area surrounding the nucleus suggesting the presence of tonoplastic structures (Fig. 2b). In open stomata the vacuole became a single compartment occupying a large part of the guard cell (Fig. 2a,b). Notably, in stomata expressing ClopHensor, the nuclear region moved to the side of the cell (Figs. 2a, 4). The distance separating the two guard cells showed that the stomata were open at the end of both the light and fusicoccin treatments (Fig. 2c). After the 3D reconstruction of the vacuolar compartment, the vacuolar volume from ClopHensor “negative staining” and VAMP711-YFP was calculated (Fig. 2; Table S1). The calculated vacuolar volumes were not statistically different (Fig. 2d; 1-way Anova p < 0.01) between the two marker lines in both closed stomata (V_vac_^ClopHensor^ = 130.9 ± 46.1 µm^3^ and V_vac_^VAMP711^ = 143.5 ± 64.8 µm^3^) and fusicoccin-open stomata (V_vac_^ClopHensor^ = 313.0 ± 67.9 µm^3^ and V_vac_^VAMP711^ = 397.7 ± 92.7 µm^3^). Moreover, the mean values of the vacuolar volume of light-opened stomata (V_vac_^ClopHensor^ = 303.5 ± 111.6 µm^3^) were similar to those induced by fusicoccin (Fig. 2d; Table S1). Interestingly, the vacuolar volume was about 2.5 times higher in open stomata compared to the closed ones. The similar values of the vacuolar volumes observed in guard cells expressing ClopHensor and VAMP711-YFP show that our “negative staining” used method is suitable to quantify the volume of the vacuole.

### 3D reconstruction of the whole guard cell subcellular volumes

In a next step, we quantified the volumetric changes of different compartments (nucleus + cytosol, vacuole and chloroplasts) of a living guard cells during stomatal opening in the same cell (Table S1). Indeed, we built 3D models of the whole guard cell divided in vacuole, nucleus-cytosol and chloroplasts (see “Material and methods”).

We imaged the same stomata closed (i.e. at the end of the dark period) and open after 3 h in the presence of fusicoccin (Fig S4). We acquired stomatal *z*-Stacks and 3D models of the whole guard cell and of the subcellular compartments were built (Fig. S3; Table S1). The chloroplasts fluorescence could be detected simultaneously with the E^2^GFP using two separated emission windows (see “Material and methods”). We therefore build 3D models of the chloroplasts and found that their volume was stable during opening being 27.4 ± 5.5 µm^3^ and 25.3 ± 3.7 µm^3^ in closed and open stomata, respectively (Fig. S4a). In contrast, the whole guard cell volume expanded from V_GC_^closed^ = 379.5 ± 50.6 µm^3^ (n = 6 guard cells) in closed stomata to V_GC_^open^ = 533.4 ± 64 µm^3^ in open stomata (n = 6 guard cells; Fig. S4a, b). This expansion corresponds to an increase of + 154 µm^3^, i.e. 1.4 times its initial volume. In the same guard cells, the vacuolar volume increased from V_Vac_^closed^ = 92.9 ± 11.6 µm^3^ to a V_Vac_^open^ = 249.5 ± 24.8 µm^3^, corresponding to a 2.7 times increase of the initial volume (Fig. S4a, b). Interestingly, the volume of the vacuole increase by + 156 µm^3^, which coincides with the volume increase of the whole-guard cell (Fig. S4). These data uncover an intriguing characteristics of stomatal opening: the whole guard cell volume increase relies on the vacuolar expansion. Indeed, in contrast with the suggestion of the 2D confocal images (Fig. 1b) the volume occupied by nucleo-cytosolic compartment remains constant during stomatal opening.

**Figure 3:**
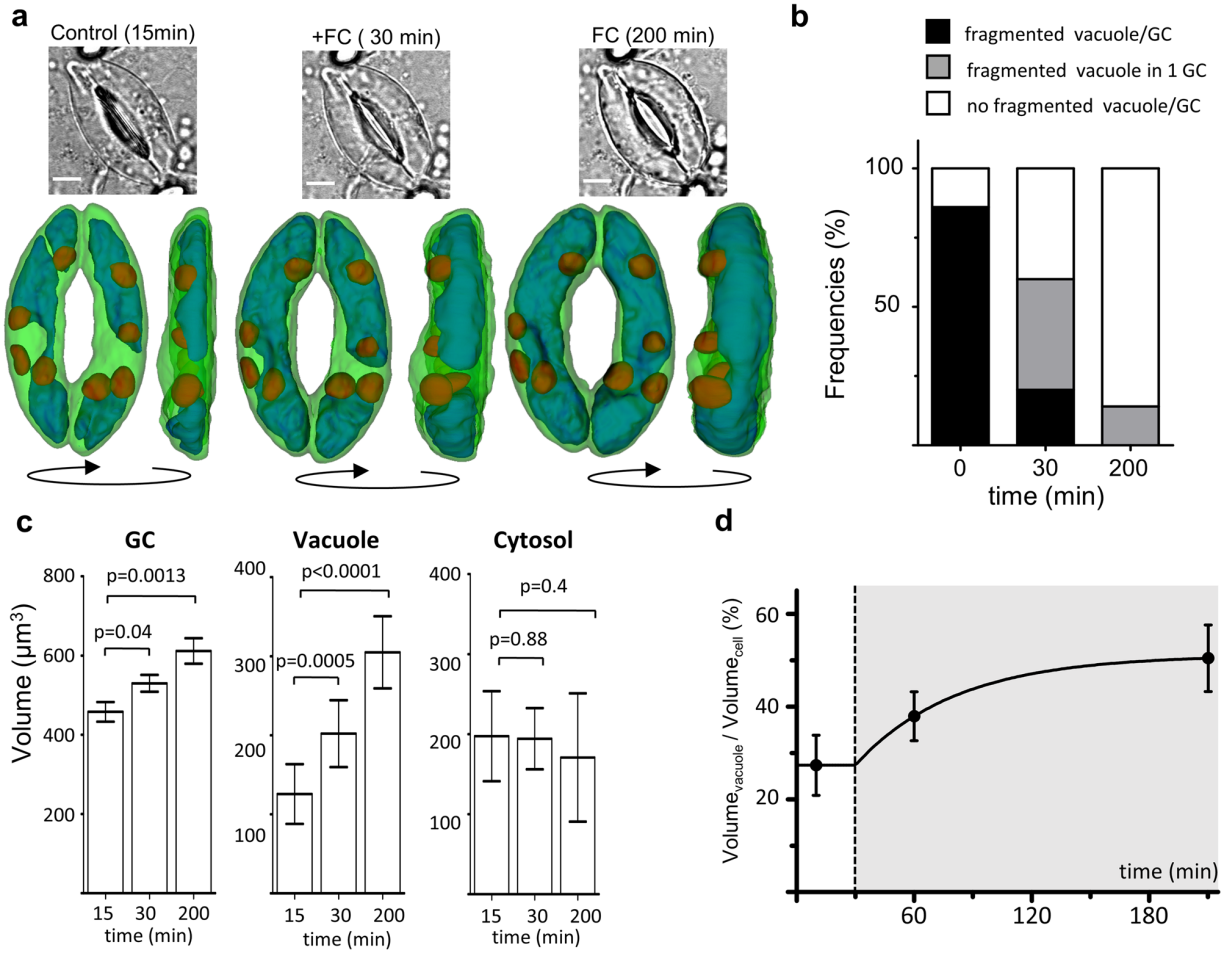
Vacuolar volume changes drive stomatal opening. **(a)** Representative transmitted light images (top) and 3D models (bottom) of single stomata before (left), 30 min (middle) and 180 min (right) after the application of 10 µM fusicoccin. The 3D models show the same stomata from the mesophyll facing side (left panel) and from the lateral side (right panel) at three time points. Blue, vacuole; green, cytosol + nucleus; red, chloroplasts. Scale bars 5 µm. (**b**) Frequencies of the different vacuolar conformations in guard cells. (**c**) Volume changes of the subcellular compartments during stomatal opening. Left, whole guard cell; center, vacuole; right*,* cytosol + nucleus (mean and S.D., n = 10 guard cells). Statistical significance was determined with a two tailed t-test, the obtained *p* values are reported. (**d**) Percentage of the guard cell volume occupied by the vacuole during opening induced by fusicoccin (grey area). Data were fitted (solid line) by a plateau followed by exponential function like in Fig. 1c (see “Material and methods”). (**a–c**) Stomatal opening was induced by exposing the guard cells to 10 µM fusicoccin at t = 30 min (vertical dashed line) for 180 min. Error bars are S.D.

We further analysed the kinetics of the subcellular changes by measuring the guard cell volumes in closed, fully open stomata, and in stomata in an intermediate state (Fig. 3; Table S1). ClopHensor fluorescence was simultaneously imaged to measure pH_cyt_, [Cl^−^]_cyt_, and for obtaining 3D reconstructions (Figs. 1, 3). We acquired *z*-Stacks of the stomata at only three time points to limit the effects of phototoxicity. We reconstructed 3D models 30 min after fusicoccin application, when the stomatal aperture was approximatively halfway from its maximum (Fig. 1c). At this time point pH_cyt_ was at its maximum (i.e. the activity of the H^+^-pumps dominate), and the [Cl^−^]_cyt_ was similar to the initial level (Fig. 1d, e). Before fusicoccin application, the vacuoles were fragmented in 86% of the guard cells, with two major vesicles at the poles of the guard cells (Fig. 3a). After 30 min in fusicoccin, the stomatal pore opening could be observed (Fig. 3a middle). The two polar vacuolar vesicles were connected at the level of the nuclear region in at least one guard cell in 80% of the stomata (Fig. 3a). Finally, 200 min after fusicoccin application, the opening was maximal and in 86% of the guard cells the vacuoles were fused in a single compartment. The vacuolar volume increased from V_Vac_^closed^ = 125.7 ± 37.8 µm^3^ to V_Vac_^30min^ = 202.0 ± 42.4 µm^3^ after 30 min, and reached V_Vac_^200min^ = 304.9 ± 45.6 µm^3^ at 200 min. In this set of experiments, the vacuole increased 2.4 times (Fig. 3b), while the whole guard cell volume increased 1.3 times between closed stomata and open at 200 min (Fig. 3b). Particularly, the volume increase of the whole guard cell and of the vacuole were similar, being + 150 µm^3^ and + 180 µm^3^ respectively. The vacuole occupied 27 ± 6% of the cell volume when stomata were closed, and 50 ± 7% when stomata were fully open (Fig. 3c). Interestingly, the nucleus-cytosolic volume (see “Material and methods”) was only marginally modified during stomatal opening with V_cyt_^closed^ = 297.5 ± 56.3 µm^3^ before fusicoccin, V_cyt_^30min^ = 294.4 ± 38.2 µm^3^ and V_cyt_^200min^ = 270.9 ± 80.2 µm^3^ after fusicoccin (Fig. 3d).

**Figure 4:**
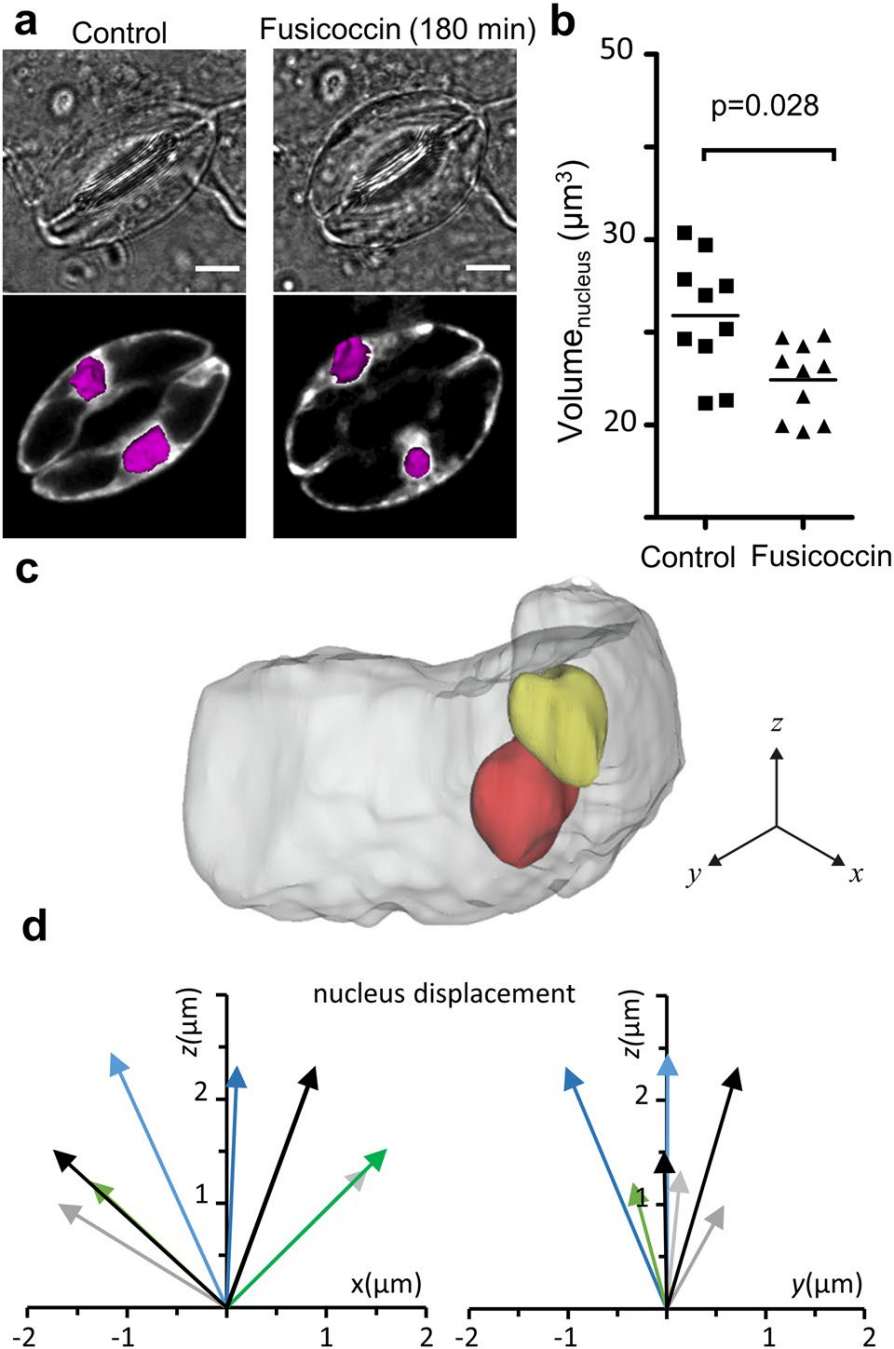
The guard cell nuclei move during stomatal opening. (**a**) Representative images of guard cell expressing ClopHensor during stomatal opening before (left column) and 180 min (right column) after applying 10 µM fusicoccin. Transmitted light (top) and fluorescence images with superimposed 3D nuclear model (bottom, purple). Scale bars 5 µm. (**b**) Nuclear volumes calculated from the 3D models, before fusicoccin application (squares; n = 10 nuclei from 5 stomata), and after 180 min in fusicoccin (triangles; n = 10 nuclei). Statistical significance was tested with a tailed t-test. (**c**) 3D model of a guard cells showing the position of the nucleus in the guard cell before (red) and after (yellow) opening. (**d**) Quantification of the position change of the nucleus (arrows) referred to the geometric center of the nucleus calculated from the 3D model of the nucleus before stomatal opening. *x, z* projection (left) and *y, z* projection (right) view of nuclei movements (n = 8 nuclei).

We noticed that the nuclear region was in a different position within the guard-cells in closed or fully open stomata (Figs. 3 and 4). In guard cells expressing ClopHensor, the nuclei could be identified as the area with the higher fluorescence intensity (Fig. 4a). In closed stomata the nuclear region was positioned in the central part of the cells, while in open stomata it moved to a peripheral area (Fig. 4a). Thus, during opening the nuclei move meanwhile the vacuole increases its volume and becomes a single compartment. We also built 3D models of the guard cells nuclei and quantified the displacement of the barycenter of the nuclei within the guard cell (Fig. 3c,d). Starting from a central position, the nuclei moved mainly in the z axis and underwent a slight decrease of the volume from 21.8 ± 2.0 to 14.9 ± 1.3µm^3^ (Fig. 4c,d).

## Discussion

The driving force regulating the stomatal aperture is the osmotic potential of the guard cells^1,2^. The osmotic potential changes stimulate water fluxes across the plasma and vacuolar membranes of guard cells, modifying the turgor pressure. In guard cells, the combination of the turgor pressure and the anisotropic properties of the cell-wall modifies the shape of the cells to open and close the stomatal pore^7,10–12^. The subcellular organization rearranges to accommodate the massive fluxes of ions and water that are required for stomatal opening. However, the morphological coordination of the different intracellular compartments was not clear so far, although it is essential for stomatal aperture regulation. Indeed, how the different subcellular compartments quantitatively participate to the change of volume of the guard cell is still to be understood.

Here, we provide a quantitative analysis of the volume dynamics of the major guard cell compartments (i.e., nucleus-cytosol, vacuole and chloroplasts) during stomatal opening. By using an original approach, we simultaneously measured, in individual living guard cell, the subcellular compartments volumes at different stages of stomatal opening. Further, we coupled volumetric measurements with the visualization of the activity of key ion transport systems (i.e. H^+^ and Cl^−^ transport; Fig. 1). We used *A. thaliana* stomata expressing ClopHensor, a biosensor allowing the simultaneously quantification of the changes of pH_cyt_ and [Cl^−^ or NO_3_^−^]_cyt_^22,24^. Additionally, we reconstructed 3D models of the whole-guard cell, the vacuole, the cytosol, and of the nucleus in the same guard cell by using the fluorescence emitted by ClopHensor (Figs. 2, 3 and 4). To induce stomatal opening we used fusiccocin^28^, a toxin directly activating the plasma membrane proton pump (Fig. 1a). This triggers H^+^ extrusion from the guard cell and hyperpolarizes the plasma membrane, mimicking stomatal opening induced by blue light^29^. Importantly, we observed overlapping vacuolar volume changes in fusicoccin- and light-induced stomatal opening (Fig. 2). The application of fusicoccin stimulated a rapid increase of the pH_cyt_ (Phase I), followed by a slow return to its initial values (Phase II; Fig. 1d). The pH_cyt_ alkalinization of Phase I depends on the activation of the plasma membrane H^+^ pumps by fusicoccin, and it coincides with the maximal pore opening rate (Fig. 1c,d). The subsequent decrease of pH_cyt_ is likely caused by the activation of secondary active transporters coupling solute transport to H^+^, such as AtCLCa^22,30^, and mediating a net flux of H^+^ into the cytosol. Thus the pH_cyt_ is controlled by several actors, including H^+^-coupled ion transport systems residing in the different cellular membranes of guard cells.

The stomatal aperture showed an increase of + 0.66 µm and of + 1.1 µm in Phase I and II, respectively (Fig. 1c). Since the pore aperture directly depends on the turgor pressure, our results indicate a higher turgor pressure increase in Phase II^10,20^. Interestingly, the vacuolar volume followed the same trend (Fig. 3). In Phase I (i.e. the first 30 min after fusicoccin application) the vacuolar volume increased by of 1.6 times with a rate of 2.54 µm^3^·min^−1^ (Fig. 3c). In Phase II (i.e. the last 120 min) the vacuole grew also 1.6 times, but showing a three times slower rate of 0.85 µm^3^·min^−1^ (Fig. 3). Since the pressure and volume are inversely related, the reduced volume growth rate in Phase II is in line with a larger turgor pressure increase in the guard cells. Accordingly, in Phase II we also observed a significant increase of the [Cl^−^]_cyt_ (Fig. 1e), indicating a higher osmotic load of the cell and thus a higher osmotic pressure.

Our results shed light on the subcellular morphological changes of the guard cells during opening. Importantly, we found that the volume of the nucleus-cytosolic compartment was constant during the whole stomatal opening process (Fig. 3). This observation is paralleled by the fact that the change in total guard cell volume quantitatively coincides with the increase in vacuolar volume. (Table S1). Taken together these data show that the vacuole provides the driving force of stomatal opening. It is interesting to note that roots cell expansion during growth similarly depends on the vacuolar size^25,31^. Recently, it was found that stomata differentiation relies on vacuolar fusion growth^19^. These data, together with ours, highlight the role of the vacuolar compartment in regulating plant cell volume. We investigated a cellular process, stomatal opening, involving fully developed cells. In both developing cells and mature guard cells, the vacuolar size and whole-cell size are tightly linked and coordinated. Nonetheless, the regulation of the stomatal aperture presents important specificities such as reversible and large turgor pressure changes in a relatively short time. These changes depend on massive uptake of solutes generating turgor pressure variations in the MPa range^7^. In these processes the whole cell is involved and the cytoskeleton plays an important role in remodelling the vacuole in both cell development and stomatal aperture^14,18,32^.

To conclude, we followed in vivo and in single cells the ion concentration dynamics of the nucleus-cytosolic compartment in parallel with the volume changes of different guard cells’ compartments. We have correlated the three-dimensional organization with the ion transport activity across the two major membranes of the guard cell: the plasma and the vacuolar membranes. We found that during stomatal opening the nucleus-cytosolic compartment remains constant while the vacuole accounts for the required whole guard cell volume increase, highlighting the importance of the vacuole in stomatal opening. More in general, our data open the question of how the relative volumes occupied by the cytosol and the vacuole within plant cells are regulated. Our data provide a step further in understanding the cellular process regulating leaf gas exchanges for both photosynthetic carbon fixation and water loss by land plants.

## Material and methods

### Plant material and growth conditions

The *Arabidopsis thaliana* transgenic lines pUBI10::ClopHensor and Col-0 pUBI10::VAMP711-YFP, in the Col-0 background, were kindly provided by Dr J. Kleine-Vehn , University of Freiburg, Germany. Seeds were vernalized 3 days in water at 4 °C in the dark before being sown in pots in a growth chamber (22 °C/20 °C, 8 h/16 h day/night photoperiod, 65% humidity, 150 μmol photons·s^−1^). GMO plants were cultivated and collected following the relevant institutional, national, and international guidelines and legislation.

### Sample preparation

Experiments were conducted on leaves from 4–5 week-old plants. The leaves were selected in order to have the same size and development. Each set of experiments were performed on stomata from at least 2 independent plants. Epidermal peel samples were prepared 1 h before light turned on in the growth chamber to have closed stomata. Epidermal strips were prepared under green light (LED Osram star classic A green) as follow: firstly, the leaf abaxial side was glued on a microscope cover slip by using a medical glue (Adapt™ 7730, Hollister, EEUU). Then the mesophyll and the adaxial side of the leaf were gently removed. To keep stomata alive, a buffer (30 mM KCl, 5 mM MES, 0.1 mM CaCl_2_, 0.1% DMSO, pH 5.7)^33^ was applied to the samples. The coverslip containing the sample was placed on a homemade perfusion chamber designed for imaging. After epidermal strips preparation, samples were kept 20 min in darkness. The buffer was regularly (every 10–15 min) refreshed either with fresh buffer (control) or with buffer solution supplemented with fusicoccin (30 mM KCl, 5 mM MES, 0.1 mM CaCl_2_, 10 μM fusicoccin, 0.1% DMSO, pH 5.7).

For experiments in Fig. 2 samples were incubated 3 h in the dark before image acquisition. In light-induced experiments, samples were incubated under light (LED-240 μM·photons^−1^·m^−2^; Ledmo^®^ LED strip Light) for 3 h. Throughout the experiments in Figs. 3 and 4, after the 20 min of incubation in the dark, samples were imaged under the microscope. Buffer solutions were applied with a manual pump system.

### Image acquisition

Stomata were imaged in vivo with an inverse confocal laser scanning microscope (CLSM) Leica SP8 (Leica, Germany) with a 63× oil objective (HCX Plan Apochromat CS 1.4 NA). Fluorescence was detected using a GaAsP Hybrid photon detector in photon counting mode. For 3D reconstruction *z*-Stacks were composed by 8 bit images with an image size of 512 × 512 pixels acquired with a 1 airy units pinhole. The optical section was 0.849 μm, the *z*-step size was 0.29 μm and the voxel size was 0.12 × 0.12 × 0.285 μm. Thus, datasets of 30–50 frames/stomata were obtained. For 3D reconstruction, the fluorescence emission of the E^2^GFP moiety of ClopHensor was detected between 500 and 550 nm after excitation at 488 nm by using an Argon laser. Chloroplasts were detected by exciting the chlorophyll at 488 nm, and the emitted fluorescence was collected in the 650–675 nm range. Transmitted light images were obtained with a PMT (Photomultiplier tube) detector. For cytosolic pH and Cl^−^ quantifications, the same CLSM setup was used with a sequential excitation mode (Excitation at 561, 488, and 458 nm). Upon excitation at 458 nm, 488 nm (Argon laser) and 561 nm (Diode Pump Solid State laser, DPSS), the emission was detected at 500–550 nm and 600–625 nm, respectively. Images were 12 bit with an image size of 256 × 256, pinhole 3 Airy units. The CLSM was driven by Las X software (Leica).

## 3D reconstruction of guard cell

### Image preparation

Image stacks were processed by using Fiji (https://imagej.net/Fiji). The pixel with the lowest value was identified with the Pixel Inspection tool, and it was subtracted to the entire stack to reduce the background. To remove noise and enhance boundaries, a median filter equal to 1 was applied to the stack. This process was common for reconstructing all subcellular compartments, except the vacuole. For the vacuole, we firstly enhanced the contours, then the brightness and contrast were automatically adjusted using the middle image each stoma as reference (Fig. S3). All processed files were saved in '.nrrd format.

### 3D segmentation

Segmentation for 3D reconstruction was performed semi-automatically by using the 3D Slicer software (https://www.slicer.org)^27^. Firstly, we assigned a single colour to each ROI (Region Of Interest) corresponding to a subcellular compartment. We semi-automatically selected the voxels forming the ROI of each compartment depending on their fluorescence intensity in the *x, y* images. For the cytosol + nucleus compartment, we selected fluorescent voxels (i.e. direct labelling method). For the vacuole, we selected non-fluorescent voxels within the cell (i.e. “negative staining”). The chloroplasts were identified based on their autofluorescence on separated *x, y* images. Finally, for each *x, y* image of the *z*-Stack the ROI of each compartment was refined taking into account the *z, x* and *z, y* projections (Fig. 2b). To build the 3D model of each compartment, the *z*-stack with the selected ROIs was processed by the Model Maker module of 3D Slicer (Fig. 2b). Files were saved in '.stl format.

### Geometrical measurements

The volume of the subcellular compartments was calculated by using the Segment Statistic tool from 3D slicer. The stomatal pore width was manually measured with Fiji from the middle image of the stomata. The geometric centre of 3D nuclear models was obtained importing the '.stl files of models in the software MeshLab (https://www.meshlab.net/). Nuclear displacement was visualized by using the 3D calculator tool from the software GeoGebra (https://www.geogebra.org/).

### Software

All software used (Fiji, 3D Slicer, MeshLab and GeoGebra) are free and open source.

## Supplementary Information

Supplementary Figures.

Supplementary Table S1.

## Data Availability

The datasets used and/or analysed during the current study available from the corresponding author on reasonable request.
