# Supplementary material for: Vacuolar control of stomatal opening revealed by 3D imaging of the guard cells
Source: Sci Rep. 2023 May 11;13:7647. doi: 10.1038/s41598-023-34273-x (PMC10175559; doi:10.1038/s41598-023-34273-x)
Supplement: Supplementary file 1 — Supplementary Figures. [file 41598_2023_34273_MOESM1_ESM.docx]

**Mirasole et al. Supplementary Figures**

**Aperture (µm)**

**time (min)**

**Figure S1. Stomata aperture kinetics.** Individual time resolved stomatal aperture kinetics over 3 hours, and after application of fusicoccin (grey area). The 11 stomata shown in the figure correspond to the stomata used in Fig 1c.

**Fig. S2 pH_cyt_ changes during Fusicoccin-induced stomatal opening measured at high frequency.** Leaf abaxial epidermis peels were obtained from Arabidopsis plants expressing ClopHensor in the cytosol before the light onset and equilibrated for 20-25 min in 30 mM KCl buffer solution before either 10 μM Fusicoccin +0.1% DMSO or control treatment (0.1% DMSO). Samples were excited at 561, 488 and 458 nm wavelengths under an Axiovert 200M ZEISS Microscope. Time 0 represents the moment of the treatment application. Ratio pH (F_488_/F_458_) at each time point upon fusicoccin treatment. Four different assays with 10 μM fusicoccin (n=47 stomata). Data are shown as mean ± SD. Fluorescence images were acquired every 15 seconds.

**Fig. S3 Image workflow for 3D reconstruction of Arabidopsis stomata**

Scheme illustrating the workflow for 3D reconstruction of the different subcellular compartments of the guard cells.

**Fig. S4 Intracellular compartments volume changes in individual guard cells.**

3D reconstruction of guard cells expressing ClopHensor.

**a** Representative confocal images of guard cells before (*left column,* 0 minutes) and after the application of Fusicoccin (*right column*, 180 minutes). Transmitted light (*top*) and confocal images superimposed to vacuole (*blue*), cell (*green*) and chloroplast (*red*) ROI or 3D models. Scale bars 5 µm. **b** Mean volume of the whole guard cell (*green* *chain line*), cytosol + nucleus (magenta *full line)* and vacuoles (*blue* *dashed line)* error bars are SD (n=6 guard cells). Cytosolic + nuclear volumes were calculated as: Volume_cytosol+nucleus_ = Volume_cell_ – Volume_vacuole_ – Volume_chloroplast_. **c** Change of percentage of the vacuolar volume with respect to the total guard cell volume. Symbols show individual guard cells and the dashed line represents the mean volume. **d** Stomata pore width measurements at two different time points. Symbols represent individual stomata, the dashed line shows the mean.
