# Supplementary material for: Vacuolar control of stomatal opening revealed by 3D imaging of the guard cells
Source: Sci Rep. 2023 May 11;13:7647. doi: 10.1038/s41598-023-34273-x (PMC10175559; doi:10.1038/s41598-023-34273-x)
Supplement: Supplementary file 2 — Supplementary Table S1. [file 41598_2023_34273_MOESM2_ESM.pdf]

Table S1

		Volume$_{guardcell}$	Volome$_{cytosol+nucleus}$	Volume$_{vacuole}$	Volume$_{chloroplasts}$	Volume$_{nucleus}$	Volume$_{vacuole}$/Volume$_{guardcell}$	Volume$_{cytosol}$/Volume$_{guardcell}$	Stomata pore width
**VAMP711-YFP$_{Fig2}$**	180 min + CS	-	-	143.5 µm3 ± 64.8	-	-	-	-	-
	180 min + FC	-	-	397.7 µm3 ± 92.7	-	-	-	-	-
	Percentage$_{(180min+FC)-(180min+CS)}$	-	-	177,10%	-	-	-	-	-
	P value$_{(180min+FC)-(180min+CS)}$	-	-	<0.0001[b]	-	-	-	-	-
**ClopHensor$_{Fig.2}$**	180 min + CS	491.6 µm3 ± 49.0	333.3 µm3 ± 23.5	130.9 µm3 ± 46.1	27.5 µm3 ± 4.8	-	26.1% ± 7.4	68.3% ± 6.9	2.6 µm ± 0.7
	180 min + FC	662.4 µm3 ± 119.6	318.6 µm3 ± 90.6	313.0 µm3 ± 67.9	30.9 µm3 ± 6.3	-	47.5% ± 7.4	47.8% ± 7.4	4.5 µm ± 0.7
	light + CS	691.4 µm3 ± 183.6	347.2 µm3 ± 75.3	303.5 µm3 ± 111.6	40.7 µm3 ± 16.8	-	43.0% ± 6.2	51.1% ± 6.2	4.0 µm ± 0.1
	Percentage$_{(180min+FC)- (180min+CS)}$	34,70%	-4,40%	139,10%	12,40%	-	-	-	73,10%
	P value$_{(180min+FC)- (180min+CS)}$	0.0001[a]	0.1749[b]	<0.0001[b]	0.1124[b]	-	<0.0001[a]	<0.0001[a]	0.0022[b]
	P value$_{(light+CS)-(180min+FC)}$	0.6506[a]	0.2602[b]	0.7508[b]	0.1572[b]	-	0.1189[a]	0.2428[a]	0.2403[b]
**ClopHensor$_{Fig.3}$**	0 min - FC	457.9µm3 ± 78.6	297.5µm3 ± 56.3	125.7µm3 ± 37.8	34.7 µm3 ± 6.3	-	27.4% ± 6.5	65.1% ± 6.6	2.4 ± 0.7
	30 min + FC	529.8µm3 ± 66.8	294.4µm3 ± 38.2	202.0µm3 ± 42.4	33.4 µm3 ± 3.3	-	37.9% ± 5.3	55.8% ± 5.2	2.9 ± 0.8
	180 min + FC	611.7µm3 ± 101.6	270.9µm3 ± 80.2	304.9µm3 ± 45.6	35.9 µm3 ± 6.7	-	50.4% ± 7.2	43.6% ± 7.3	4.3 ± 0.3
	Percentage$_{(30min-FC)-(0min+FC)}$	15,70%	-1%	60,70%	-3,70%	-	-	-	20,80%
	Percentage$_{(180min+FC)-(30min+FC)}$	15,50%	-8%	50,90%	7,50%	-	-	-	48,30%
	Percentage$_{(180min-FC)-(0min+FC)}$	33,60%	-8,90%	142,60%	3,50%	-	-	-	79,20%
	P value$_{(30min-FC)-(0min+FC)}$	0.0081[a]	0.8164[a]	<0.0001[a]	0.4958[a]	-	0.0008[a]	0.0026[a]	0.5476[b]
	P value$_{(180min+FC)-(30min+FC)}$	0.0176[a]	0.3553[a]	<0.0001[a]	0.0912[a]	-	0.0003[a]	0.0005[a]	0.0159[b]
	P value$_{(180min-FC)-(0min+FC)}$	0.0008[a]	0.2959[a]	<0.0001[a]	0.5998[a]	-	<0.0001[a]	<0.0001[a]	0.0079[b]
**ClopHensorFig.4**	0 min - FC	-	-	-	-	21.8 µm3 ± 2.0	-	-	-
	180 min + FC	-	-	-	-	14.9 µm3 ± 1.3	-	-	-
	Percentage$_{(180min+FC)-(0min-FC)}$	-	-	-	-	-31.7%	-	-	-
	P value$_{(180min+FC)-(0min-FC)}$	-	-	-	-	0.0280[a]	-	-	-
**ClopHensor$_{Fig.S3}$**	0 min - FC	379.5 µm3 ± 50.6	259.2 µm3 ± 48.6	92.9 µm3 ± 11.6	27.4 µm3 ± 5.5	-	24.8 % ± 4.3	67.9% ± 4.3	3.1 ± 0.5
	180 min + FC	533.4 µm3 ± 63.7	258.6 µm3 ± 60.2	249.5 µm3 ± 24.8	25.3 µm3 ± 3.7	-	47.2% ± 5.8	48.0% ± 6.2	4.4 ± 0.2
	Percentage$_{(180min+FC)-(0min-FC)}$	40,60%	-0,20%	168,60%	-7,70%	-	-	-	41,90%
	P value$_{(180min+FC)-(0min-FC)}$	0.0022[b]	1.0000[b]	0.0022[b]	0.3939[b]	-	0.0022[b]	0.0022[b]	0.1000[b]

a=t test; b= Mann-Whitney; CS=control solution; FC= Fusicoccin.
